# Building country capacity to sustain NTD programs and progress: A call to action

**DOI:** 10.1371/journal.pntd.0008565

**Published:** 2020-10-08

**Authors:** Yao Sodahlon, David A. Ross, Carol McPhillips-Tangum, Joni Lawrence, Rachel Taylor, Deborah A. McFarland, Alison Krentel, Chukwuma Anyaikea, Wilfred Etienne Batcho, Clarisse Bougouma, Andreia de Pádua Careli Dantas, Neeraj Dhingra, Marilia Massangaie Guambe, Khairiah Ibrahim, Ibrahim J. Kargbo-Labour, Gabriel K. Matwale, Farah-Nelhy Momprevil, Merita Antonia Armindo Monteiro, Georges Barthelemy Nko'Ayissi, Wyckliff Peter Omondi, Luc Herman Randrianirina, Batchiri A. Salissou, Harland R. Schuler, Alzira Segunda Silva do Rosário, Laston Douglas Sitima, Kwamy M. Togbey, Bucumi Victor, Mwelecele Ntuli Malecela

**Affiliations:** 1 Mectizan Donation Program, Task Force for Global Health, Decatur, Georgia, United States of America; 2 Task Force for Global Health, Decatur, Georgia, United States of America; 3 CMT Consulting, LLC, Decatur, Georgia, United States of America; 4 Office of Corporate Responsibility, Merck & Co., Inc., Kenilworth, New Jersey, United States of America; 5 Rollins School of Public Health, Emory University, Atlanta, Georgia, United States of America; 6 Bruyère Research Institute, Ottawa, Canada; 7 Department of Public Health, Neglected Tropical Diseases Division, Federal Ministry of Health, Abuja, Nigeria; 8 Programme National de Lutte Contre les Maladies Transmissibles, Ministère de la Santé, Cotonou, Republic of Bénin; 9 Programme National de Lutte Contre les Maladies Tropicales Négligées, Ministère de la Santé, Ouagadougou, Burkina Faso; 10 Health Surveillance Secretariat, Ministry of Health, Brasília/Federal District, Brazil; 11 National Vector Borne Diseases Control Programme, Ministry of Health and Family Welfare, New Delhi, India; 12 Departamento de Outras Doenças Transmissíveis, Ministério da Saúde, Maputo City, Mozambique; 13 Disease Control Division, Ministry of Health, Putrajaya, Malaysia; 14 Neglected Tropical Diseases Programme, Ministry of Health and Sanitation, Freetown, Sierra Leone; 15 Neglected Tropical Diseases Control Program, Ministry of Health, Kampala, Uganda; 16 Nationaux de Malaria et de Filariose Lymphatique, Ministere de la Sante Publique et de la Population, Port-au-Prince, Haiti; 17 Department of Communicable Disease Control, Ministry of Health, Dili, Timor-Leste; 18 Neglected Tropical Disease Program, Ministry of Health, Yaoundé, Cameroon; 19 Division of Vector Borne and Neglected Tropical Diseases, Ministry of Health, Nairobi, Kenya; 20 Division of Emergencies and Control of Communicable Diseases, Ministry of Public Health, Antananarivo, Madagascar; 21 Onchocerciasis and Lymphatic Filariasis Program, Ministry of Health, Niamey, Niger; 22 Servicio Autónomo Instituto de Biomedicina, Ministerio del Poder Popular para la Salud, Caracas, Venezuela; 23 Programa Doenças Tropicais Negligenciadas, Ministério de Saúde, Agua Grande, São Tomé e Príncipe; 24 Onchocerciasis Control Program, Ministry of Health, Lilongwe, Malawi; 25 Neglected Tropical Disease Program, Ministry of Health and Public Hygiene, Lomé, Togo; 26 National Integrated Programme to Combat Neglected Tropical Diseases and Blindness in Burundi, Ministry of the Public Health and the Fight Against AIDS, Bujumbura, Burundi; 27 Department of Control of Neglected Tropical Diseases, World Health Organization, Geneva, Switzerland; Swiss Tropical and Public Health Institute, SWITZERLAND

Neglected tropical diseases (NTDs) affect more than 1 billion people worldwide and disproportionately trap some of the world’s most vulnerable and marginalized populations into a cycle of poverty and disease [1]. Although NTDs place a heavy burden on public health, particularly among low- and middle-income countries, progress has been made over the past few decades. Since the London Declaration in 2012, about 31 countries have eliminated at least one NTD [2]. Much of the progress toward the control and elimination of NTDs is due to three factors: (1) Countries have committed to reaching more people with NTD interventions, (2) the pharmaceutical industry has donated billions of treatments for NTDs, and (3) philanthropists have provided generous funding to implement NTD interventions [3].

The impetus for many donors to join the fight against NTDs came in 1987, a time when Merck & Co, Inc., (known as MSD outside the United States and Canada) announced that it would donate its ivermectin (Mectizan) for as long as needed to treat onchocerciasis (oncho) in all endemic countries. As a result of Merck’s commitment and the partnerships developed to distribute ivermectin, mass drug administration (MDA) has emerged as the leading strategy to control and eliminate oncho [4]. The success of MDA and these new partnerships catalyzed additional pharmaceutical companies to donate other drugs intended to combat NTDs. As the momentum to control and eliminate NTDs grew, pharmaceutical companies joined other donors, nongovernmental organizations (NGOs), and NTD endemic countries in signing the London Declaration on NTDs in 2012. The fifth progress report called on endemic countries to increase their financial and political commitment to NTDs, scale-up programs to reach all at risk, and invest domestic resources in fighting NTDs [5].

Countries are contemplating how to increase domestic investments to sustain their NTD programs and maintain their progress. During a Coalition for Operational Research on NTDs (COR-NTD) meeting in July 2019, NTD program managers expressed the need for technical assistance with advocacy and domestic fundraising. In this forum, countries were able to engage with one another to identify and share promising strategies to increase domestic support and resources to combat NTDs [6]. These discussions are timely as global goals, and directives increasingly call for better integration of NTD programming into universal healthcare for financial sustainability over the long term. The anticipated economic impacts of the COVID-19 pandemic, including the possibility of reduced donor funding for NTDs, could make it even more challenging to build country capacity to sustain NTD programs.

The WHO’s NTD Roadmap for 2021 to 2030 encourages three fundamental shifts in the approach to fighting NTDs: (1) increasing accountability through the use of impact indicators, (2) moving to more cross-cutting NTD programs, and (3) building sustainability by ensuring greater country ownership of NTD programs [7]. To achieve the third goal, countries will need to have national plans related to NTDs and contribute domestic funding to tackle these diseases.

Despite recommendations to combat NTDs by increasing country ownership and domestic spending, implementing such recommendations will require a shift in country practices. We have little empirical knowledge of how endemic countries are preparing for this type of shift. As a result, the Mectizan Donation Program and the Task Force for Global Health conducted an online survey of NTD program managers working on oncho and lymphatic filariasis (LF) to better understand country-level practices and challenges related to increasing domestic spending to sustain NTD interventions. Of the 44 NTD program managers who were invited, 34 (77.3%) participated in the survey between October and December of 2019.

The survey results confirm that nearly all (97.1%) of oncho and LF endemic countries rely heavily on external funding and other resources to support their NTD elimination activities. Despite heavy reliance on donor resources, contingency planning (if donor resources are reduced or withdrawn) is uncommon. Only about one-half of survey respondents (54.5%) have had internal discussions about how they would fund NTD elimination activities domestically if donor resources were reduced or withdrawn. Among those who have had these discussions, only about one-third (38.9%) have a plan for generating or advocating for increased domestic investment in NTD elimination.

**Fig 1:**
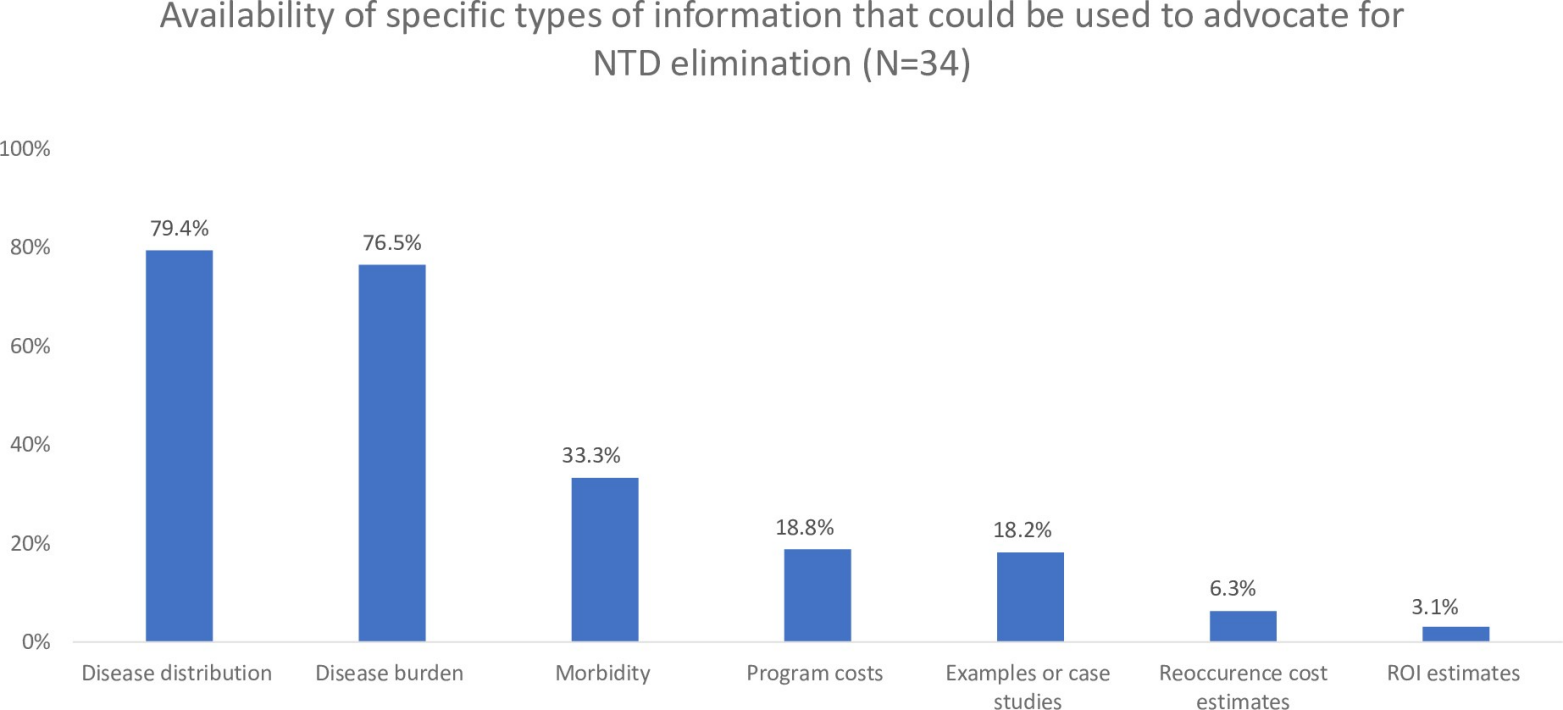
Availability of specific types of information that could be used to advocate for NTD elimination (*n* = 34).

NTD program managers face many challenges to serving as effective advocates. Nearly all respondents reported needing information related to disease burden, disease distribution, and morbidity (100%, 97.1%, and 100%, respectively). The vast majority of respondents also noted the importance of having information about the costs to conduct or sustain NTD elimination programs, estimates of the costs that would be incurred by the country if disease reoccurs, and estimates of the return-on-investment (ROI) related to NTD elimination (97.0%, 93.9%, and 90.6%, respectively). However, much of this needed information is incomplete or simply unavailable. Financial and economic information seem to be especially lacking, as the majority of survey respondents indicated that information about NTD program costs, reoccurrence costs, and ROI are incomplete or unavailable (75.0%, 87.6%, and 78.2%, respectively; Fig 1). Only about one-third of respondents (35.3%) reported that their country’s ministry of finance plays a key role in advocating for domestic resources to sustain NTD elimination activities.

The most common challenges that NTD program managers anticipate or face when advocating for NTD resources include the competing priorities of decision-makers, insufficient budget and resources, low mortality associated with NTDs, lack of effective partnerships, and perception that NTD elimination is already well-resourced. When asked about the types of resources that could be used to help advocate domestically for NTD resources, several respondents reported needing country-specific information about the burden of NTDs that could be shared with decision-makers. Respondents were also asked to indicate their interest in and willingness to use a calculator or similar type of tool that could provide information about what a country could gain by investing in NTD elimination and what could happen without such investment. In response, 73.5% indicated they would use such a tool, and an additional 11.8% said they would consider using it.

In light of our survey findings, growing interest in increasing country ownership and domestic spending for NTD elimination activities, and realization that COVID-19 will pose new challenges to NTD programs, we propose that it is time to develop and implement strategies to build country capacity to sustain NTD programs and their progress. The recommendations cited in the WHO Roadmap provide the necessary impetus to prompt countries toward financial sustainability, but recommendations are not sufficient to help prepare for this shift. Therefore, we argue that a series of actions should be taken to build country capacity to sustain NTD programs and progress.

First, a comprehensive approach to building advocacy capacity within countries is needed. Additional information should be gathered from endemic countries to further assess their needs and preferences for the types of guidance and resources that could be developed to build their capacity to be effective advocates. NTD program managers must be included in all discussions and decisions to ensure that the developed approach meets their needs. The capacity building efforts should also be expanded to local advocacy organizations who promote other public health issues.

Second, a set of advocacy resources should be developed. Based on additional input from endemic countries and other possible end-users, these resources should be designed to enhance the knowledge, skills, and self-efficacy of NTD program managers and other health leaders to effectively advocate for increased domestic investment in NTD programs. It will be incumbent upon those who develop the resources to adequately train and support countries in their use.

Third, consideration should be given to enhancing the financial literacy of NTD program managers and other health leaders who will need to serve as advocates for domestic funding and resources. To be effective, these individuals will need to “speak the same language” of colleagues and counterparts in the Ministry of Finance and other organizations in which the focus is on economics and finance.

Fourth, assistance should be provided to NTD program managers to assist them in accessing and packaging needed information on disease burden, distribution, cost, and ROI. Without the ability to access and use this type of information, program managers will remain challenged to effectively partner with ministries of finance to expand domestic funding for NTD programs.

Finally, a broad and comprehensive approach to building country capacity to sustain NTD programs and their progress will require diverse and dedicated partnerships. Within countries, partnerships must be developed and/or strengthened between those who work in the ministries of health and those who work in the ministries of finance. Within the community of donors, NGOs, and other conveners, partners should coalesce around the need to develop and implement strategies to build country capacity to sustain their NTD programs.

We are at a unique moment in time. Countries are motivated to increase ownership of and domestic spending for their NTD programs. They are asking for guidance. The community of donors, NGOs, and other stakeholders involved in supporting endemic countries must seize upon this moment by recognizing and acting upon the need to build country capacity to sustain NTD programs and the progress they have made over time.
